# Impact of donor age on liver transplant outcomes in patients with hepatocellular carcinoma: analysis of the SRTR database

**DOI:** 10.1186/s12876-021-01786-6

**Published:** 2021-04-30

**Authors:** Jie Zhou, Zhichao Huang, Zheng Chen, Fangshen Xu, Rongliang Tong, Shusen Zheng

**Affiliations:** 1 Division of Hepatobiliary and Pancreatic Surgery, Department of Surgery, First Affiliated Hospital, Zhejiang University, School of Medicine, Hangzhou, China; 2 NHC Key Laboratory of Combined Multi-Organ Transplantation, Hangzhou, China; 3 Key Laboratory of the Diagnosis and Treatment of Organ Transplantation, Research Unit of Collaborative Diagnosis and Treatment For Hepatobiliary and Pancreatic Cancer, Chinese Academy of Medical Sciences (2019RU019), Hangzhou, China; 4 Key Laboratory of Organ Transplantation, Research Center for Diagnosis and Treatment of Hepatobiliary Diseases, Hangzhou, 310003 Zhejiang Province China

**Keywords:** Liver transplantation, Donor age, Hepatocellular carcinoma, Outcome, SRTR

## Abstract

**Background:**

Donor age is an important predictor for liver transplant recipients. Studies have not fully explored its impact on transplant outcomes in hepatocellular carcinoma (HCC) patients as well as its involvement in tumor recurrence.

**Methods:**

HCC patients who received liver transplants during 2010–2017 from the Scientific Registry of Transplant Recipients database were included. The recipients were divided into four groups based on donor age: I (≤ 34 years), II (35–49 years), III (50–64 years), and IV (≥ 65 years). Transplant outcomes, including the overall survival (OS), tumor recurrence, and risks, were evaluated.

**Results:**

A total of 13,276 HCC recipients were included in this study. Statistical significant differences were observed in OS among the four groups. The best 5-year survival was 76.0% in group I, followed by 73.5% in group II, 72.8% in group III, and 69.2% in group IV (*P* < 0.001). However, the liver-specific survival did not differ among these groups (*P* = 0.260). Donor age was found to be the independent predictor of OS after adjusting for other variables (*P* < 0.001, ref. group I; 1.087 (0.979–1.208) for group II, *P* = 0.119; 1.124 (1.015–1.246) for group III, *P* = 0.025; 1.395 (1.215–1.602) for group IV, *P* < 0.001). In subgroup analysis, OS was significantly different in recipients with hepatitis C virus (HCV), but there was no significant difference for recipients with hepatitis B virus (HBV), alcoholic liver diseases and nonalcoholic steatohepatitis (NASH). The post-transplant cumulative tumor recurrence rates were similar among the four groups (*P* = 0.382).

**Conclusions:**

Older donor age was associated with decreased OS but not liver-specific survival as well as post-transplant tumor recurrence in HCC recipients. Donor age also had different effects in patients with different underlying liver diseases.

**Supplementary Information:**

The online version contains supplementary material available at 10.1186/s12876-021-01786-6.

## Background

Hepatocellular carcinoma (HCC), the fourth most common cause of cancer-related death worldwide, accounts for more than 700,000 deaths annually and its incidence continues to increase [1–3]. Only a minority of HCC patients are feasible candidates for hepatectomy, which is due to the fact that most patients are diagnosed at the advanced stages and often presented with poor liver function. Tumor recurrence after hepatectomy also remains high. Liver transplantation is the only curative option to treat HCC patients. However, due to the disparity between the large number of candidates and the relative shortage of donor livers, many patients dropped out of the waiting list before they could match with a feasible donor graft.

Marginal donor usage has been suggested to expand the donor pool. Older donors are the most commonly used marginal donors. The number of older liver donors is increasing due to the ageing population [4]. However, there are concerns regarding the application of old liver grafts in clinical practice, as liver tissue from older donors would undergo a series of morphological and physiological changes, making it more vulnerable to ischemia–reperfusion injury (IRI) during transplantation [5]. These pathophysiological changes would lead to adverse effects on recipient prognosis. Feng et al. evaluated the effects of donor-related variables on transplant outcomes and demonstrated that donor age was an independent predictor of overall survival (OS) [6]. And they have created a model known as the donor risk index (DRI) to stratify the risk of transplant outcomes based on a series of donor variables. However, with an increasing application of liver grafts from older donors and improved surgical techniques as well as perioperative managements, a number of studies have recently shown the safety of use of old donor livers, even using livers from octogenarian donors [4, 7].

Despite this, the effect of donor age on liver transplantation specifically in HCC patients has not yet been fully elucidated. Tumor recurrence after liver transplantation is an important concern in those patients. We have already established recipient selection criteria so that donors are matched with the most feasible patients to obtain favorable post-transplant outcomes [8, 9]. However, current practical experience with regard to how donor variables such as donor age could affect transplant outcomes, including tumor recurrence, in HCC patients is limited. Although previous studies have demonstrated the association between donation after cardiac death (DCD) and post-transplant mortality in HCC recipients, they have not focused on tumor recurrence [10]. Orci et al. has previously evaluated the effect of donor characteristics on tumor recurrence after liver transplantation based on recipients through 2004 to 2011 from the Scientific Registry of Transplant Recipients (SRTR) database [11]. Yet policies for treatment of HCC have changed during this time, with improvements in liver transplantation and increased use of marginal donors. Therefore, it is important to re-evaluate the donor characteristics on transplant outcomes in HCC patients in this setting.

Here, we use renewed data from the SRTR database to evaluate the effect of donor age on liver transplant outcomes, especially OS and tumor recurrence, in patients with HCC.

## Methods

This study used data from the Scientific Registry of Transplant Recipients (SRTR). The SRTR data system includes data on all donor, wait-listed candidates, and transplant recipients in the US, submitted by the members of the Organ Procurement and Transplantation Network (OPTN). The Health Resources and Services Administration (HRSA), U.S. Department of Health and Human Services provides oversight to the activities of the OPTN and SRTR contractors. The data reported here have been supplied by the Hennepin Healthcare Research Institute (HHRI) as the contractor for the Scientific Registry of Transplant Recipients (SRTR). The interpretation and reporting of these data are the responsibility of the author(s) and in no way should be seen as an official policy of or interpretation by the SRTR or the U.S. Government [12]. Organs from executed prisoners were not used in this study. The protocol for the present study was in accordance with the Declaration of Helsinki and was approved by the Ethics Committee of the First Affiliated Hospital, College of Medicine, Zhejiang University, China (approval number 2019-1020).

**Fig. 1:**
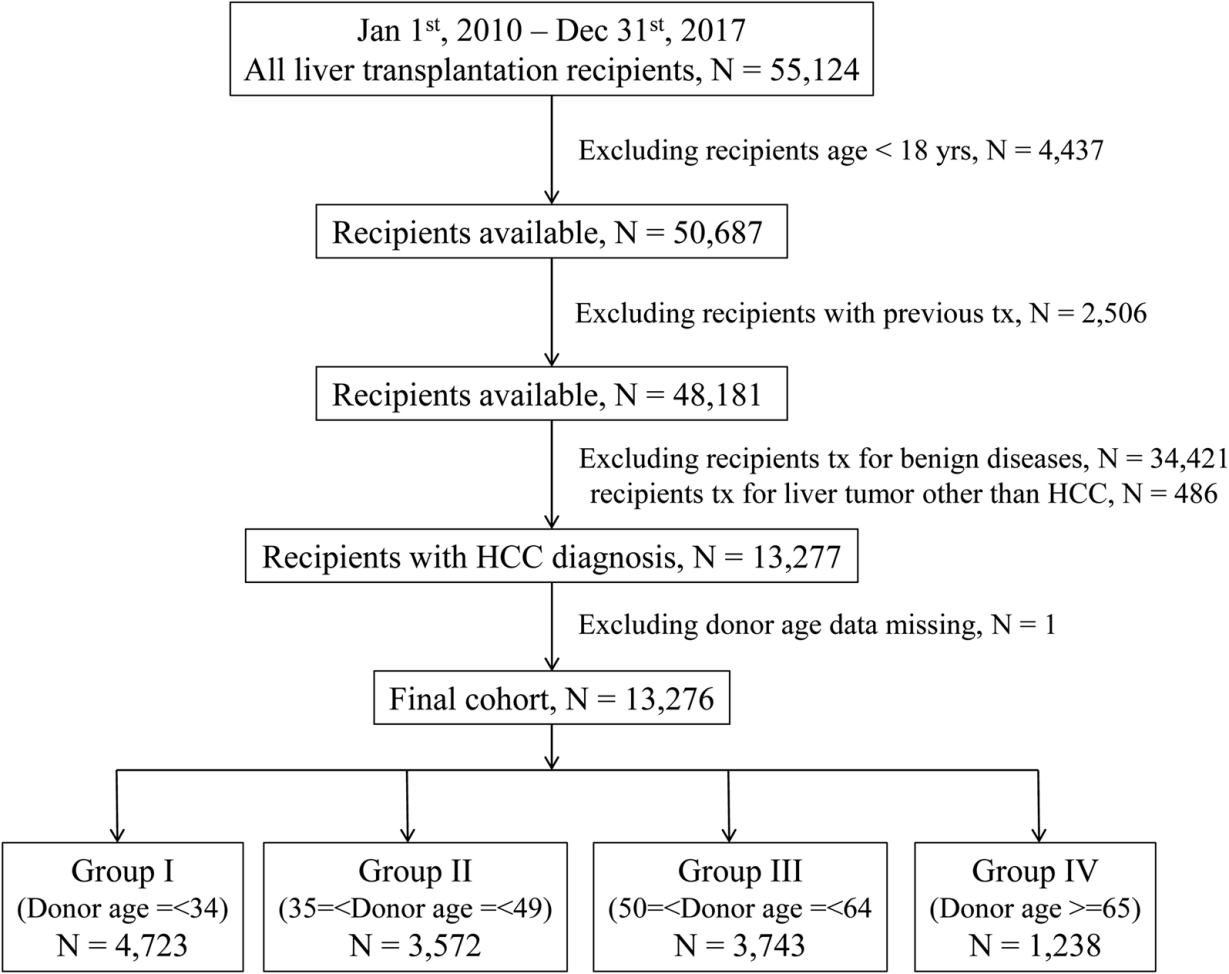
Flow chart of the patient selection process

We included patients with HCC who received liver transplantation from January 1, 2010, to December 31, 2017. The inclusion criteria were: recipients ≥ 18 years old, with a primary diagnosis of “hepatocellular carcinoma” or “hepatoma” at transplant. Patients with a previous liver transplantation, those who received transplant for benign liver disease or liver tumor other than HCC, and those < 18 years old were excluded from this study. Finally a total of 13,276 recipients were included in the current study. Patients were followed up to death or the end of the study on 1st March, 2019. Figure 1 illustrates the patient selection flowchart.

To assess how donor age affects transplant outcomes, we divided recipients into four groups according to donor age: group I, donor age ≤ 34 years (N = 4723); group II, donor age 35–49 years (N = 3572); group III, donor age 50–64 years (N = 3743); group IV, donor age ≥ 65 years (N = 1238). The donor and recipient data as well as tumor characteristics were compared among the four groups.

For long-term outcomes, we first compared post-transplant OS among four groups, which was evaluated from the time of LT and defined recipient death as the endpoint. OS was also assessed based on recipient underlying liver diseases including hepatitis C virus (HCV), hepatitis B virus (HBV), alcoholic liver diseases and nonalcoholic steatohepatitis (NASH). Liver-specific survival was also analyzed, which was measured from the date of LT to date of liver-related death or last follow-up. Patient cause of death, including graft failure, cardiovascular/cerebrovascular disease, organ failure, hemorrhage, infection and HCC recurrence, were compared among the four groups, first within 30 days after transplantation and then for the whole population. The post-transplant HCC recurrence was compared among the four groups, and the definition was in accordance with the report by Samoylova et al. [13]. The detailed identification procedure was performed in accordance with that described in the study by Orci et al. [11].

### Statistical analysis

Baseline characteristics were compared using one-way ANOVA for continuous variables and the chi-square test for binomial variables. OS and liver-specific survival was assessed by the Kaplan–Meier method and log-rank test was used to compare differences among the groups. Univariate analysis was used to identify potential predictors for OS, and those with *P* < 0.05 were further analyzed in the multivariable analysis using the Cox proportional hazard ratios (HRs) model. Time-dependent effects were evaluated based on Schoenfeld’s residuals, and cubic spline functions were introduced in the model [14]. The cumulative tumor recurrence rates were evaluated using the competing risk model. A two-tailed P value < 0.05 was considered statistically significant. All the analyses were conducted with SPSS version 22.0 (IBM, Armonk, NY, United States) and R for Windows (version 4.0.2).

## Results

### Baseline characteristics

The median follow-up time was 36 months (interquartile range, 18–60 months) for the entire study population. Donor characteristics including donor height, weight, race, ABO blood type, sex, cause of death, deceased donor type (DCD or not) significantly differed among the four groups. For recipient characteristics, the recipient age, race, ABO blood type, underlying liver disease, height, weight, cold ischemia time, body mass index (BMI), laboratory model for end-stage liver disease (MELD) score, albumin, bilirubin, international normalized ratio (INR), creatinine and immunosuppression maintenance with tacrolimus, cyclosporin, mycophenolate mofetil and steroids at discharge were all statistically different among the four groups. However, the recipient warm ischemia time, pre-transplant sodium level and immunosuppression induction (with anti-CD25 or thymoglobulin) as well as sirolimus maintenance at discharge were comparable among the four groups. Analysis of tumor characteristics revealed that the pre-transplant treatment (including transarterial chemoembolization (TACE), radiofrequency ablation (RFA), chemotherapy, cryoablation, surgery), number of tumors, largest tumor diameter, sum of tumor diameters, tumor staging, and pre-transplant α-fetoprotein (AFP) level were all similar among four groups. Table 1 presents a summary of the data in detail.

**Table 1:** Baseline characteristics

	Group I (Donor age ≤ 34, N = 4723)	Group II (Donor age 35–49, N = 3572)	Group III (Donor age 50–64, N = 3743)	Group IV (Donor age ≥ 65, N = 1238)	*P* value
*Donor variables*					
Race					< 0.001
White	3051 (64.6%)	2269 (63.5%)	2378 (63.5%)	872 (70.4%)	
Black or African American	814 (17.2%)	647 (18.1%)	757 (20.2%)	188 (15.2%)	
Asian	82 (1.7%)	96 (2.7%)	114 (3.0%)	51 (4.1%)	
Hispanic/Latino	725 (15.4%)	524 (14.7%)	465 (12.4%)	123 (9.9%)	
Other	51 (1.1%)	36 (1.0%)	29 (0.8%)	4 (0.3%)	
ABO					< 0.001
A	1752 (37.1%)	1361 (38.1%)	1346 (36.0%)	467 (37.7%)	
B	667 (14.1%)	490 (13.7%)	506 (13.5%)	129 (10.4%)	
O	2129 (45.1%)	1584 (44.3%)	1796 (48.0%)	620 (50.1%)	
AB	175 (3.7%)	137 (3.8%)	95 (2.5%)	22 (1.8%)	
Gender					< 0.001
M	3226 (68.3%)	2048 (57.3%)	2013 (53.8%)	619 (50.0%)	
F	1497 (31.7%)	1524 (42.7%)	1730 (46.2%)	619 (50.0%)	
Cause of death					< 0.001
Anoxia	1812 (39.4%)	1262 (36.5%)	1045 (28.3%)	202 (16.3%)	
Cerebrovascular/stroke	429 (9.3%)	1285 (37.1%)	2003 (54.2%)	815 (65.9%)	
Head trauma	2234 (48.6%)	801 (23.1%)	586 (15.8%)	205 (16.6%)	
CNS tumor	27 (0.6%)	25 (0.7%)	12 (0.3%)	1 (0.1%)	
Other	94 (2.0%)	88 (2.5%)	52 (1.4%)	14 (1.1%)	
DCD	494 (10.5%)	302 (8.5%)	153 (4.1%)	1 (0.1%)	< 0.001
Height (cm)	172.58 ± 11.56	171.48 ± 10.11	170.32 ± 10.13	168.83 ± 10.18	< 0.001
Weight (kg)	78.71 ± 20.49	86.30 ± 21.54	84.02 ± 20.75	79.67 ± 18.30	< 0.001
*Recipient variables*					
Gender					< 0.001
M	3599 (76.2%)	2854 (79.9%)	2929 (78.3%)	909 (73.4%)	
F	1124 (23.8%)	718 (20.1%)	814 (21.7%)	329 (26.6%)	
Age	59.62 ± 7.31	59.49 ± 7.16	59.89 ± 6.75	61.55 ± 6.64	< 0.001
Race					< 0.001
White	3074 (65.1%)	2400 (67.2%)	2524 (67.4%)	825 (66.6%)	
Black or African American	500 (10.6%)	373 (10.4%)	332 (8.9%)	85 (6.9%)	
Asian	332 (7.0%)	203 (5.7%)	253 (6.8%)	109 (8.8%)	
Hispanic/Latino	753 (15.9%)	552 (15.5%)	590 (15.8%)	201 (16.2%)	
Other	64 (1.4%)	44 (1.2%)	44 (1.2%)	18 (1.5%)	
ABO					< 0.001
A	1733 (36.7%)	1344 (37.6%)	1328 (35.5%)	462 (37.3%)	
B	684 (14.5%)	509 (14.2%)	498 (13.3%)	131 (10.6%)	
O	2062 (43.7%)	1532 (42.9%)	1780 (47.6%)	609 (49.2%)	
AB	244 (5.2%)	187 (5.2%)	137 (3.7%)	36 (2.9%)	
Underlying liver disease					< 0.001
HCV	3147 (66.6%)	2389 (66.9%)	2372 (63.4%)	556 (44.9%)	
HBV	329 (7.0%)	228 (6.4%)	271 (7.2%)	152 (12.3%)	
Alcohol	412 (8.7%)	347 (9.7%)	366 (9.8%)	184 (14.9%)	
NASH	396 (8.4%)	309 (8.7%)	363 (9.7%)	171 (13.8%)	
Other	439 (9.3%)	299 (8.4%)	371 (9.9%)	175 (14.1%)	
Height (cm)	172.39 ± 10.04	173.06 ± 9.85	172.75 ± 9.99	170.91 ± 9.96	< 0.001
Weight (kg)	85.07 ± 18.82	86.72 ± 18.52	86.75 ± 18.66	84.40 ± 17.84	< 0.001
Warm ischemia time (min)	40.11 ± 20.31	40.29 ± 18.80	40.86 ± 19.69	40.61 ± 21.13	0.646
Cold ischemia time (h)	6.2 1 ± 2.65	6.06 ± 2.52	6.21 ± 2.50	6.02 ± 2.21	0.006
BMI	28.55 ± 5.47	29.00 ± 7.73	29.04 ± 6.24	28.83 ± 5.40	0.001
MELD	15.40 ± 8.77	15.40 ± 8.53	14.74 ± 7.97	14.01 ± 6.83	< 0.001
Albumin (g/dl)	3.21 ± 0.69	3.23 ± 0.69	3.25 ± 0.70	3.27 ± 0.67	0.01
Bilirubin (mg/dl)	4.05 ± 7.32	3.91 ± 7.01	3.56 ± 6.39	3.21 ± 5.62	< 0.001
INR	1.53 ± 0.73	1.55 ± 0.79	1.51 ± 0.74	1.46 ± 0.68	0.002
Creatinine (mg/dl)	1.23 ± 1.13	1.19 ± 1.01	1.12 ± 0.86	1.04 ± 0.62	< 0.001
Sodium (mmol/L)	137.12 ± 4.51	137.16 ± 4.42	137.28 ± 4.40	137.17 ± 4.45	0.389
Treatment					
TACE	2577 (64.9%)	1990 (65.5%)	2140 (65.6%)	757 (69.0%)	0.093
RFA	489 (12.3%)	341 (11.2%)	365 (11.2%)	108 (9.8%)	0.107
Chemotherapy	123 (3.1%)	89 (2.9%)	116 (3.6%)	32 (2.9%)	0.482
Cryoablation	12 (0.3%)	13 (0.4%)	14 (0.4%)	2 (0.2)	0.54
Surgery	39 (1.0%)	31 (1.0%)	43 (1.3%)	18 (1.6%)	0.207
Tumor staging					0.592
Within Milan	3680 (97.4%)	2795 (96.8%)	3026 (97.1%)	1023 (97.2%)	
Beyond Milan	99 (2.6%)	92 (3.2%)	91 (2.9%)	30 (2.8%)	
Tumor nubmer	1.24 ± 0.55	1.24 ± 0.54	1.24 ± 0.56	1.25 ± 0.55	0.197
Largest tumor diameter (cm)	1.54 ± 1.40	1.57 ± 1.61	1.57 ± 1.40	1.57 ± 1.44	0.463
Sum of tumor diameters (cm)	1.88 ± 1.81	1.91 ± 1.98	1.91 ± 1.80	1.94 ± 1.88	0.381
AFP value (ng/ml)	8 (4, 24)	8 (4, 27)	8 (4, 24)	7 (4, 20)	0.781
*Immunosuppression*					
Induction					0.096
Anti-CD25	826	599	633	211	
Thymoglobulin	398	334	290	84	
Anti-CD25 + Thymoglobulin	10	5	9	0	
Maintenance					
Tacrolimus	3179	2309	2392	751	< 0.001
Cyclosporin	117	93	78	16	0.036
Sirolimus	154	118	119	46	0.834
Mycophenolate mofetil	2236	1733	1694	570	0.036
Steroids	1709	1244	1248	459	0.021

AFP, α-fetoprotein; BMI, body mass index; CNS, central nervous system; DCD, donation after cardiac death; HBV, hepatitis B virus; HCV, hepatitis C virus; INR, international normalized ratio; MELD, model for end-stage liver disease; NASH, nonalcoholic steatohepatitis; RFA, radiofrequency ablation; TACE, transarterial chemoembolization

### OS

**Fig. 2:**
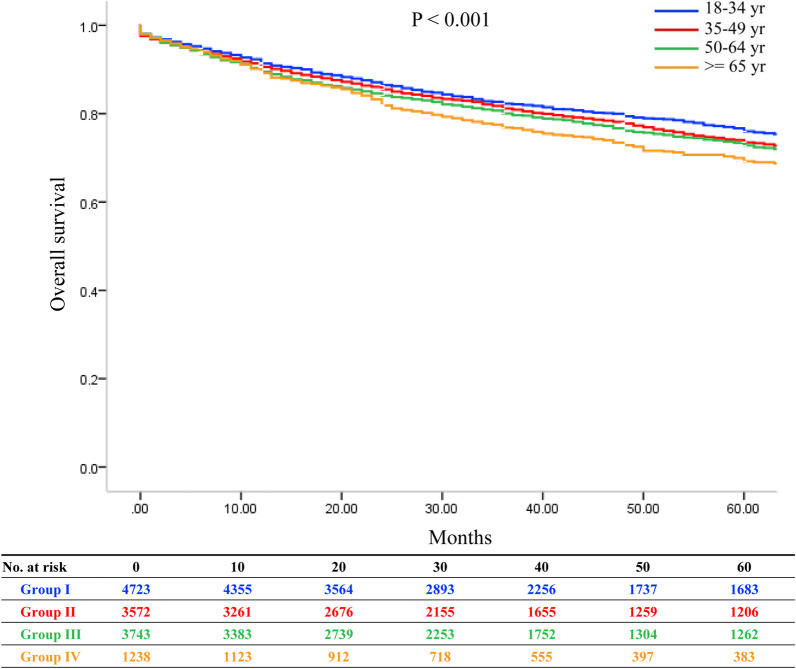
Overall survival of HCC recipients categorized by different donor age groups

We compared OS among the four groups. The 1-, 3-, and 5-year OS were 91.4%, 82.3%, and 76.0% in group I; 90.6%, 81.2%, and 73.5% in group II; 89.5%, 80.0%, and 72.8% in group III; and 89.2%, 76.8%, and 69.2% in group IV, respectively (*P* < 0.001; Fig. 2). We also compared the OS between two groups at a time, and observed that the survival of group I recipients was better than that of the other three groups (group I vs. group II, *P* = 0.049; group I vs. group III, *P* = 0.002; group I vs. group IV, *P* < 0.001). The survival of group II recipients was comparable to that of group III (*P* = 0.308) but significantly better than that of group IV recipients (*P* = 0.004). The survival of group III recipients was also better than group IV (*P* = 0.038). We also compared OS after excluding patients whose follow-up time was < 24 months, and observed similar outcomes (Additional file 1: Table S1 and Additional file 2: Figure S1). For liver-specific survival, no differences were observed among four age groups (Additional file 1: Table S2 and Additional file 3: Figure S2).

No differences were observed for cause of death within post-transplant 30 days. However, recipients had higher incidence rates of graft failure (*P* = 0.009), organ failure (*P* = 0.002) and infection (*P* = 0.001) in the older donor age group during follow-up (Table 2).

**Table 2:** Cause of mortality in liver transplant recipients with donors from different age groups

	Mortality within 30 days					Overall mortality				
	Group I (Donor age = < 34, N = 132)	Group II (Donor age 35–49, N = 113)	Group III (Donor age 50–64, N = 105)	Group IV (Donor age > = 65, N = 35)	*P* value	Group I (Donor age = < 34, N = 4723)	Group II (Donor age 35–49, N = 3572)	Group III (Donor age 50–64, N = 3743)	Group IV Donor age (> = 65, N = 1238)	*P* value
Graft failure	15 (11.4%)	18 (15.9%)	13 (12.4%)	5 (14.3%)	0.749	61 (1.3%)	60 (1.7%)	82 (2.2%)	27 (2.2%)	0.009
Cardiovascular/cerebrovascular	45 (34.1%)	38 (33.6%)	38 (36.2%)	9 (25.7%)	0.73	128 (2.8%)	98 (2.7%)	97 (2.6%)	33 (2.7%)	0.98
Organ failure	20 (15.2%)	14 (12.4%)	13 (12.4%)	5 (14.3%)	0.906	112 (2.4%)	98 (2.7%)	109 (2.9%)	54 (4.4%)	0.002
Hemorrhage	9 (6.8%)	14 (12.4%)	10 (9.5%)	3 (8.6%)	0.521	21 (0.4%)	26 (0.7%)	19 (0.5%)	4 (0.3%)	0.224
Infection	19 (14.4%)	13 (11.5%)	16 (15.2%)	7 (20%)	0.631	72 (1.5%)	67 (1.9%)	97 (2.6%)	34 (2.7%)	0.001
HCC recurrence	0	0	0	0	N/A	157 (3.3%)	108 (3.0%)	109 (2.9%)	43 (3.5%)	0.62
Others	24 (18.2%)	16 (14.2%)	15 (14.3%)	6 (17.1%)	0.797	449 (9.5%)	358 (10.0%)	376 (10.0%)	135 (10.9%)	0.503

HCC, Hepatocellular carcinoma

**Fig. 3:**
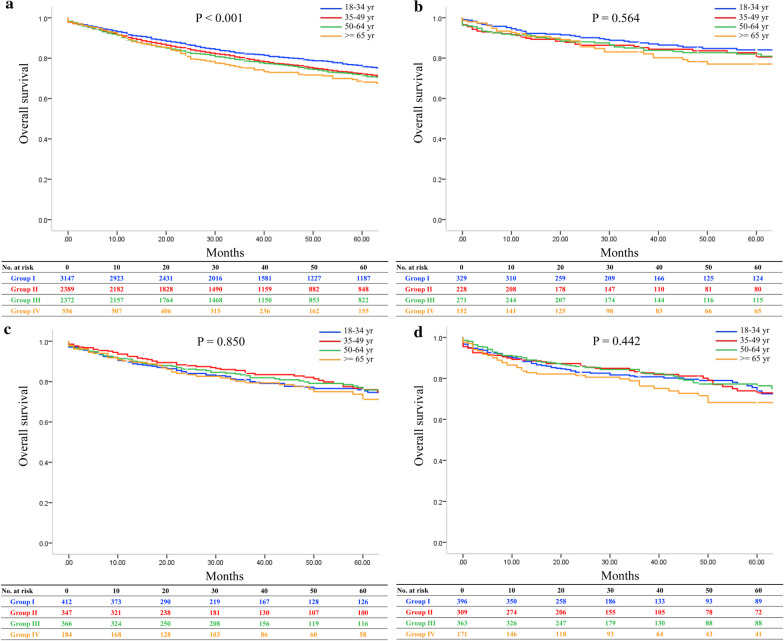
Overall survival of HCC recipients in different donor age groups according to underlying liver diseases: **a** HCV; **b** HBV; **c** alcoholic liver diseases; **d** NASH

Next, we analyzed the survival according to recipient underlying liver diseases. The OS significantly differed in recipients with HCV, with 1-, 3-, and 5-year OS rates at 91.4%, 82.1%, and 75.8% in group I; 90.2%, 79.8%, and 72.1% in group II; 89.1%, 78.6%, and 71.4% in group III; and 89.3%, 75.1%, and 68.1% in group IV, respectively (*P* < 0.001; Fig. 3a). However, there were no significant differences in the survival rates among the four groups of patients in subsets with HBV, alcoholic liver diseases and NASH. The 1-, 3-, and 5-year OS rates were 93.3%, 87.5%, and 84.1% in group I; 90.3%, 85.2%, and 80.6% in group II; 91.5%, 85.0%, and 82.0% in group III; and 92.1%, 83.1%, and 77.1% in group IV in recipients with HBV (*P* = 0.564; Fig. 3b). For recipients with alcoholic liver diseases, the 1-, 3-, and 5-year OS rates were 89.7%, 81.0%, and 75.4% in group I; 92.4%, 84.1%, and 76.0% in group II; 91.2%, 83.0%, and 75.8% in group III; and 90.2%, 79.4%, and 71.2% in group IV (*P* = 0.850; Fig. 3c). The OS rates in recipients with NASH were 88.3%, 80.8%, and 73.9% in group I; 89.0%, 82.7%, and 72.9% in group II; 90.0%, 83.0%, and 76.4% in group III; and 83.5%, 76.3%, and 68.2% in group IV (*P* = 0.442; Fig. 3d). OS rates were also analyzed after excluding patients whose follow-up time was < 24 months, and similar outcomes were observed (Additional file 1: Table S3 and Additional file 4: Figure S3). Liver-specific survival was also analyzed and there were no differences among four groups in underlying liver diseases (Additional file 1: Table S4 and Additional file 5: Figure S4).

**Table 3:** Univariate analysis of predictors for recipient overall survival

	HR (95% CI)	*P* value
*Donor variables*		
Age (reference Group I)		< 0.001
Group II	1.097 (1.000–1.203)	0.050
Group III	1.153 (1.053–1.262)	0.002
Group IV	1.319 (1.164–1.493)	< 0.001
Race (reference White)		0.778
Black or African American	1.008 (0.917–1.108)	0.868
Asian	0.983 (0.783–1.235)	0.885
Hispanic/Latino	1.011 (0.910–1.124)	0.832
Other	0.750 (0.483–1.165)	0.200
ABO (reference A)		0.123
B	0.925 (0.824–1.039)	0.189
O	1.053 (0.974–1.139)	0.196
AB	0.961 (0.782–1.181)	0.706
Gender	1.005 (0.969–1.042)	0.781
Cause of death (reference Anoxia)		0.002
Cerebrovascular/stroke	1.112 (1.019–1.214)	0.017
Head trauma	0.960 (0.874–1.055)	0.397
CNS tumor	0.452 (0.215–0.950)	0.036
Other	0.955 (0.722–1.263)	0.747
DCD	1.052 (0.916–1.208)	0.476
Height (cm)	0.997 (0.994–1.000)	0.085
Weight (kg)	1.000 (0.999–1.002)	0.587
*Recipient variables*		
Age	1.018 (1.013–1.024)	< 0.001
Gender (F vs. M)	0.972 (0.931–1.015)	0.195
Race (reference White)		< 0.001
Black or African American	1.177 (1.050–1.320)	0.005
Asian	0.756 (0.645–0.885)	< 0.001
Hispanic/Latino	0.852 (0.767–0.946)	0.003
Other	0.965 (0.706–1.320)	0.826
ABO (reference A)		0.226
B	0.946 (0.844–1.061)	0.345
O	1.044 (0.965–1.130)	0.28
AB	0.927 (0.773–1.111)	0.41
Underlying liver disease (reference HCV)		< 0.001
HBV	0.687 (0.586–0.804)	< 0.001
Alcohol	0.905 (0.796–1.029)	0.126
NASH	1.014 (0.890–1.154)	0.839
Other	1.038 (0.922–1.169)	0.535
Height (cm)	1.003 (1.000–1.007)	0.071
Weight (kg)	1.000 (0.998–1.002)	0.849
Warm ischemia time (min)	1.000 (0.998–1.003)	0.674
Cold ischemia time (h)	0.999 (0.986–1.013)	0.915
BMI	0.997 (0.990–1.003)	0.303
MELD	1.016 (1.012–1.020)	< 0.001
Albumin (g/dl)	0.893 (0.848–0.940)	< 0.001
Bilirubin (mg/dl)	1.013 (1.008–1.017)	< 0.001
INR	1.084 (1.040–1.131)	< 0.001
Creatinine (mg/dl)	1.086 (1.061–1.111)	< 0.001
Sodium (mmol/L)	0.991 (0.983–0.999)	0.034
Tumor staging (beyond vs. within Milan)	1.420 (1.160–1.738)	0.001
Tumor nubmer	1.113 (1.043–1.187)	0.001
Largest tumor diameter (cm)	1.065 (1.045–1.086)	< 0.001
Sum of tumor diameters (cm)	1.061 (1.043–1.079)	< 0.001
AFP value (ng/ml) > 400	1.928 (1.599–2.325)	< 0.001
Pretransplant treatment	0.998 (0.912–1.091)	0.958
Immunosuppression Induction (reference anti-CD25)		0.514
Thymoglobulin	1.074 (0.921–1.253)	0.362
Anti-CD25 + Thymoglobulin	1.370 (0.612–3.064)	0.444
Maintenance at discharge		
Tacrolimus	0.884 (0.819–0.954)	0.002
Cyclosporine	1.127 (0.912–1.391)	0.269
Sirolimus	1.157 (0.969–1.382)	0.108
Mycophenolate mofetil	0.915 (0.852–0.982)	0.014
Steroids	0.898 (0.832–0.968)	0.005

AFP, α-fetoprotein; BMI, body mass index; CNS, central nervous system; DCD, donation after cardiac death; HBV, hepatitis B virus; HCV, hepatitis C virus; INR, international normalized ratio; MELD, model for end-stage liver disease; NASH, nonalcoholic steatohepatitis

### Univariate analysis for OS

We then performed univariate analysis to identify potential risk factors for recipient OS. Donor characteristics, including donor age and cause of death; recipient characteristics, including recipient age, race, underlying liver diseases, pre-transplant laboratory MELD score, albumin, bilirubin, INR, creatinine, and sodium levels; tumor characteristics including number of tumors, largest tumor diameter, sum of tumor diameters, tumor staging, and pre-transplant AFP level; immunosuppression maintenance with tacrolimus, mycophenolate mofetil and steroid at discharge were all found to be significantly associated with the OS. Table 3 presents this information in greater detail.

### Multivariable analysis for OS

The multivariable Cox regression analysis showed that donor age, recipient age, race, underlying liver diseases, pre-transplant MELD score, creatinine, sum of tumor diameters, AFP level, immunosuppression maintenance with tacrolimus and steroid at discharge were all independent predictors of OS. Table 4 presents this information in greater detail. Further univariate as well as multivariable analysis for OS were also performed for those patients with follow-up time ≥ 24 months and detailed information was shown in Additional file 1: Table S5 and S6.

**Table 4:** Multivariable analysis of predictors for recipient overall survival

	HR (95% CI)	*P* value
Donor age (reference Group I)		< 0.001
Group II	1.087 (0.979–1.208)	0.119
Group III	1.124 (1.015–1.246)	0.025
Group IV	1.395 (1.215–1.602)	< 0.001
Recipient age	1.021 (1.014–1.027)	< 0.001
Recipient race (reference White)		0.013
Black or African American	1.141 (1–1.301)	0.050
Asian	0.878 (0.726–1.062)	0.181
Hispanic/Latino	0.882 (0.783–0.993)	0.038
Other	1.178 (0.85–1.633)	0.326
Underlying liver diseases (reverence HCV)		0.011
HBV	0.724 (0.596–0.879)	0.001
Alcoholic liver diseases	0.935 (0.803–1.088)	0.383
NASH	0.878 (0.748–1.029)	0.108
Other	0.992 (0.864–1.138)	0.904
MELD	1.013 (1.006–1.019)	< 0.001
Recipient serum creatinine (mg/dl)	1.051 (1.008–1.095)	0.019
Sum of tumor diameters (cm)	1.052 (1.033–1.072)	< 0.001
AFP (≥ 400 ng/ml vs. < 400 ng/ml)	1.993 (1.648–2.412)	< 0.001
Tacrolimus maintenance at discharge	0.911 (0.835–0.994)	0.035
Steroids maintenance at discharge	0.762 (0.696–0.835)	< 0.001

AFP, α-fetoprotein; HBV, hepatitis B virus; HCV, hepatitis C virus; MELD, model for end-stage liver disease; NASH, nonalcoholic steatohepatitis

### Time-dependent effect of donor age on OS

**Fig. 4:**
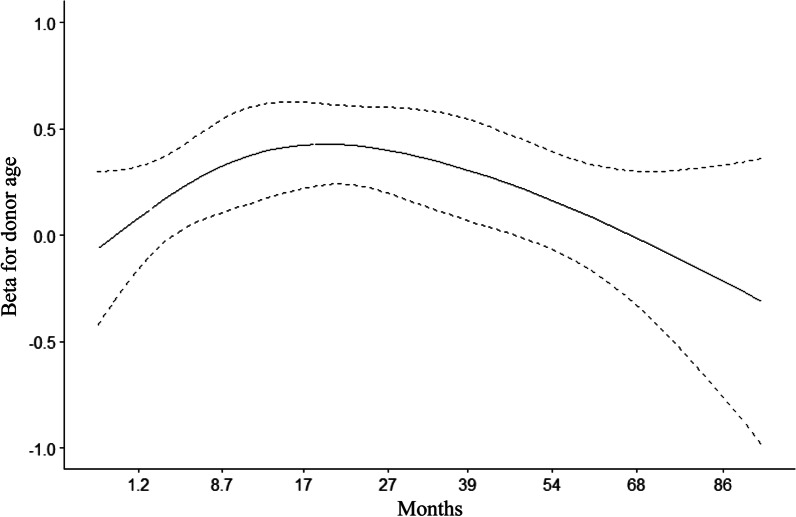
Cox-derived estimates of the time-dependent hazard ratio of donor age for OS in HCC recipients

We specifically analyzed the time-dependent HR of donor age on the recipient OS and observed that the HR remained generally stable during the follow-up, but it tended to increase in the early transplant months and decrease over the years (Fig. 4). This suggested that although donor age was a constant independent predictor of decreased OS, the negative effect of older donor age may tend to decrease over time.

### Post-transplant tumor recurrence

A total of 567 recipients suffered HCC recurrence in the post-transplant period. Patients with tumor recurrence had significantly inferior OS compared to those without recurrence (5-year survival 8.7% vs. 77.8% respectively, *P* < 0.001, Additional file 6: Figure S5).

The general tumor recurrence rates were comparable among the four age groups (218 (4.6%) in group I, 142 (4.0%) in group II, 150 (4.0%) in group III and 57 (4.6%) in group IV, *P* = 0.375). The median time to recurrence after transplantation was 22 months (interquartile range, 10–33 months) in group I, 22.5 months (12–35 months) in group II, 21.5 months (12–35.5 months) in group III and 22 months (11–37 months) in group IV. To further analyze the cumulative tumor recurrence rates, we introduced the competing risk model. We observed that the cumulative recurrence rates in the four groups were also similar, with a 5-year cumulative recurrence rate of 5.54% in group I, 4.98% in group II, 4.80% in group III and 5.84% in group IV (*P* = 0.382, Additional file 7: Figure S6).The cumulative recurrence rate after excluding patients whose follow-up time < 24 months were also compared among four groups and showed similar trends (*P* = 0.368).

As no difference was observed in terms of recurrence rates among the groups, additional Cox regression model and competing regression model were not used. However, we further investigated the time-dependent effect of donor age on tumor recurrence in a sensitive analysis. The effect of donor age also remained generally stable in the early transplant years, and showed a decreasing trend over the long-term follow-up period (Additional file 8: Figure S7).

## Discussion

In this study, we demonstrated that the OS differed in HCC liver transplant recipients categorized according to different donor ages, whereas donor age had no effect on post-transplant tumor recurrence. With regard to underlying liver diseases, there were differences in OS in HCV recipients, but not in recipients with HBV, alcoholic liver diseases or NASH.

The impact of donor age on transplant outcomes has been evaluated for decades with controversies surrounding the safety and feasibility of grafts from older donors. Some studies revealed the association between older donor age and decreased recipient survival while others shared successful experience of the use of septuagenarian and even octogenarian donors [4, 6, 7, 15–17]. Also, little is known about how donor age affects tumor recurrence in recipients with HCC. Studies have shown that liver grafts from older donors would be more vulnerable to IRI [5]. And increased IRI was found to be associated with tumor recurrence [18, 19]. Thus, there is a potential that older donor age can lead to higher tumor recurrence rate after transplantation.

We found that there were significant statistical differences in OS among four age groups, while no difference was observed with regard to tumor recurrence. Indeed the actual survival differences were minimal among four groups, especially at post-transplant 1 year. The significant statistical difference in OS might be due to the large cohort of patients included in this study, which might not necessarily mean clinical relevance. Moreover, the liver-specific survival was similar among four groups, which might indicate that older donor age had minimal impact on liver graft to affect long-term survival in HCC recipients.

We also observed the differential effects of donor age on recipients with different underlying liver diseases. In patients with HCV, older donor age was associated with decreased OS, while in patients with HBV, alcoholic liver diseases and NASH, no differences in OS were observed among four groups. This result is in accordance with that reported by Lake et al. [20], who reported that donor age did not have any effect on post-transplant outcomes in recipients with HBV. Their study also showed that transplants from donors > 60 years were associated with decreased survival in patients with underlying liver diseases other than HBV and HCV. However, they did not sub-classify these diseases, which we think is important in the current transplant practice, as the number of patients with NASH has been increasing in recent years and it has become one of the leading indications of liver transplantation [21, 22]. Therefore, our preliminary results of impact of donor age on different underlying liver diseases in HCC population is worth validation and further exploration to study the different causes of liver diseases in transplant recipients.

Recipient age was also found to be an independent predictor of OS in HCC recipients, which was in accordance with previous literature [23–26]. Patients with HCC are generally older than other candidates on the waiting list [22, 25, 27]. Factors including cardiovascular diseases, functional status such as frailty as well as higher extra-hepatic tumor risks are all the negative factors associated with older recipient age, which may lead to adverse outcomes. Moreover, sum of tumor diameters and pre-transplant AFP level were independent predictors of OS, reflecting the importance of the candidate selection process in HCC patients, which we should not only evaluate the general status of the recipient but also tumor characteristics using HCC selection criteria, such as the Milan Criteria etc. [8, 28]. We also observed that immunosuppression maintenance with tacrolimus and steroids at discharge were independent predictors. However, the beneficial role of sirolimus on HCC shown in previous literature was not observed in our study [29, 30]. This might be due to the relatively low proportion of sirolimus use in our cohort. Also, we could not evaluate the effect of immunosuppression doses and immunosuppression maintenance duration during the follow-up due to the limited data availability in the current database. Further studies with more detailed information on immunosuppression regime and a larger cohort are needed.

Our study has several limitations. First, as it is based on a large dataset, we could not analyze the possible confounding variables such as tumor biology as assessed by Edmondson-Steiner grade and microvascular invasion, which are important predictors for overall survival and tumor recurrence in HCC patients. Also, variables related to new therapeutic advances in HCC treatment, such as the use of targeted therapies before or after transplantation, were also not available in the database. This is important because the prognosis of HCC patients has considerably improved in recent years with the development of targeted therapy and immunotherapy. Secondly, as our study included recipients from 2010 to 2017, there was a potential that a relatively short follow-up would confound the true risk of tumor recurrence after transplantation, especially with the currently ongoing development of new treatments for HCC, which might delay or diminish the recurrence of HCC. Nonetheless, our study presents the largest study to date to evaluate the effect of donor age on HCC patients based on the latest transplantation recipient cohort. In the future, prospective studies with long follow-up time need to be designed to investigate the effect of donor age in a more detailed manner to expand the donor pool and benefit more candidates on the waiting list.

## Conclusions

Our study demonstrated that although older donor age was associated with statistical inferior OS in transplant recipients with HCC, the actual survival differences were minimal. Moreover, older donor age was not correlated with decreased liver-specific survival as well as post-transplant tumor recurrence, which might indicate that it had minimal impact on long-term outcomes. For different underlying liver diseases, older donor age was associated with inferior OS in recipients with HCV but with no influence in recipients with HBV, alcoholic liver diseases or NASH. These findings may be useful for clinicians in decision-making with regards to marginal donor allocation and recipient selection to achieve favorable transplant outcomes.

## Supplementary Information

**Additional file 1:**  Table S1 - S6.

**Additional file 2: Figure S1.** Overall survival of HCC recipients with post-transplant follow-up time ≥ 24 months categorized by different donor age groups.

**Additional file 3: Figure S2.** Liver-specific survival of HCC recipients categorized by different donor age groups.

**Additional file 4: Figure S3.** Overall survival of HCC recipients with post-transplant follow-up time ≥ 24 months in different donor age groups according to underlying liver diseases: **a** HCV; **b** HBV; **c** alcoholic liver diseases; **d** NASH.

**Additional file 5: Figure S4.** Liver-specific survival of HCC recipients in different donor age groups according to underlying liver diseases: **a** HCV; **b** HBV; **c** alcoholic liver diseases; **d** NASH.

**Additional file 6: Figure S5.** Overall survival between HCC recipients with and without post-transplant tumor recurrence.

**Additional file 7: Figure S6.** Cumulative HCC recurrence rates categorized by different donor age groups.

**Additional file 8: Figure S7.** Cox-derived estimates of the time-dependent hazard ratio of donor age for HCC recurrence after liver transplantation.

## Data Availability

The data that support the findings of this study are available from the Scientific Registry of Transplant Recipients database, but restrictions apply to the availability of these data, which were used under license for the current study, and so are not publicly available. Data are however available from the authors upon reasonable request and with permission of the Scientific Registry of Transplant Recipients database. The protocol for the present study was in accordance with the Declaration of Helsinki and was approved by the Ethics Committee of the First Affiliated Hospital, College of Medicine, Zhejiang University, China (Approval Number 2019-1020).

## Abbreviations

AFP: α-Fetoprotein
BMI: Body mass index
CI: Confidence interval
CNS: Central nervous system
DCD: Donation after cardiac death
DRI: Donor risk index
HBV: Hepatitis B virus
HCC: Hepatocellular carcinoma
HCV: Hepatitis C virus
HR: Hazard ratios
INR: International normalized ratio
IRI: Ischemia–reperfusion injury
MELD: Model for end-stage liver disease
NASH: Nonalcoholic steatohepatitis
OPTN: Organ Procurement and Transplantation Network
OS: Overall survival
RFA: Radiofrequency ablation
SRTR: Scientific Registry of Transplant Recipients
TACE: Transarterial chemoembolization
