# Supplementary material for: Impact of donor age on liver transplant outcomes in patients with hepatocellular carcinoma: analysis of the SRTR database
Source: BMC Gastroenterol. 2021 Apr 30;21:195. doi: 10.1186/s12876-021-01786-6 (PMC8086097; doi:10.1186/s12876-021-01786-6)
Supplement: Supplementary file 1 — Additional file 1: Table S1 - S6. [file 12876_2021_1786_MOESM1_ESM.docx]

Additional file 1: Table S1. Overall survival of HCC recipients with post-transplant follow-up time longer than 24 months

|  | 1-year | 3-year | 5-year |
| --- | --- | --- | --- |
| Group I (N = 4,077) | 90.0% | 81.1% | 74.9% |
| Group II (N = 3,102) | 89.2% | 79.9% | 72.4% |
| Group III (N = 3,233) | 87.9% | 78.6% | 71.5% |
| Group IV (N = 1,080) | 87.7% | 75.6% | 68.1% |

Additional file 1: Table S2. Liver-specific survival of HCC recipients

|  | 1-year | 3-year | 5-year |
| --- | --- | --- | --- |
| Group I (N = 4,723) | 98.1% | 95.6% | 93.8% |
| Group II (N = 3,572) | 98.1% | 95.5% | 93.5% |
| Group III (N = 3,743) | 97.7% | 95.1% | 93.1% |
| Group IV (N = 1,283) | 98.0% | 94.2% | 92.4% |

Additional file 1: Table S3. Overall survival of HCC recipients with post-transplant follow-up time longer than 24 months according to underlying liver diseases

| HCV | 1-year | 3-year | 5-year |
| --- | --- | --- | --- |
| Group I (N = 2,769) | 90.5% | 81.1% | 74.9% |
| Group II (N = 2,129) | 89.1% | 78.7% | 71.2% |
| Group III (N = 2,097) | 87.7% | 77.4% | 70.3% |
| Group IV (N = 483) | 87.8% | 73.9% | 67.0% |
| HBV | 1-year | 3-year | 5-year |
| Group I (N = 285) | 92.3% | 86.6% | 83.2% |
| Group II (N = 204) | 89.2% | 84.1% | 79.6% |
| Group III (N = 235) | 90.2% | 83.8% | 80.8% |
| Group IV (N = 140) | 91.4% | 82.5% | 76.5% |
| Alcoholic liver disease | 1-year | 3-year | 5-year |
| Group I (N = 341) | 87.7% | 79.1% | 73.7% |
| Group II (N = 272) | 90.4% | 82.3% | 74.4% |
| Group III (N = 291) | 89.0% | 81.0% | 74.0% |
| Group IV (N = 151) | 88.1% | 77.5% | 69.5% |
| NASH | 1-year | 3-year | 5-year |
| Group I (N = 314) | 85.4% | 78.1% | 71.4% |
| Group II (N = 244) | 86.1% | 80.0% | 70.6% |
| Group III (N = 291) | 87.6% | 80.8% | 74.3% |
| Group IV (N = 125) | 81.1% | 74.0% | 66.2% |

Abbreviations: HBV, Hepatitis B virus; HCV, Hepatitis C virus; NASH, Nonalcoholic steatohepatitis.

Additional file 1: Table S4. Liver-specific survival of HCC recipients according to underlying liver diseases

| HCV | 1-year | 3-year | 5-year |
| --- | --- | --- | --- |
| Group I (N = 3,147) | 97.9% | 95.2% | 93.1% |
| Group II (N = 2,389) | 97.9% | 94.9% | 93.1% |
| Group III (N = 2,372) | 97.3% | 94.9% | 92.6% |
| Group IV (N = 556) | 97.2% | 91.7% | 90.1% |
| HBV | 1-year | 3-year | 5-year |
| Group I (N = 329) | 98.1% | 96.9% | 96.3% |
| Group II (N = 228) | 98.1% | 96.5% | 94.8% |
| Group III (N = 271) | 98.1% | 94.7% | 94.7% |
| Group IV (N = 152) | 99.3% | 98.3% | 94.8% |
| Alcoholic liver disease | 1-year | 3-year | 5-year |
| Group I (N = 412) | 98.7% | 97.5% | 97.5% |
| Group II (N = 347) | 98.2% | 96.9% | 94.2% |
| Group III (N = 366) | 99.1% | 97.3% | 96.7% |
| Group IV (N = 184) | 98.3% | 95.6% | 95.6% |
| NASH | 1-year | 3-year | 5-year |
| Group I (N = 396) | 98.4% | 96.5% | 95.4% |
| Group II (N = 309) | 98.2% | 97.1% | 95.9% |
| Group III (N = 363) | 98.0% | 96.3% | 95.6% |
| Group IV (N = 171) | 99.4% | 97.2% | 97.2% |

Abbreviations: HBV, Hepatitis B virus; HCV, Hepatitis C virus; NASH, Nonalcoholic steatohepatitis.

Additional file 1: Table S5. Univariate analysis of predictors for recipient overall survival whose follow-up time was longer than 24 months

|  | HR (95% CI) | P value |
| --- | --- | --- |
| Donor variables |  |  |
| Age (reference Group I) |  | < 0.001 |
| Group II | 1.094 (0.998 - 1.2) | 0.056 |
| Group III | 1.153 (1.053 - 1.262) | 0.002 |
| Group IV | 1.313 (1.159 - 1.487) | < 0.001 |
| Race (reference White) |  | 0.804 |
| Black or African American | 1.009 (0.918 - 1.108) | 0.859 |
| Asian | 0.989 (0.788 - 1.242) | 0.925 |
| Hispanic/Latino | 1.016 (0.915 - 1.129) | 0.765 |
| Other | 0.762 (0.491 - 1.184) | 0.227 |
| ABO (reference A) |  | 0.089 |
| B | 0.922 (0.821 - 1.035) | 0.167 |
| O | 1.057 (0.977 - 1.143) | 0.167 |
| AB | 0.958 (0.779 - 1.178) | 0.684 |
| Gender | 1.006 (0.97 - 1.043) | 0.74 |
| Cause of Death (reference Anoxia) |  | 0.004 |
| Cerebrovascular/stroke | 1.075 (0.985 - 1.173) | 0.106 |
| Head trauma | 0.931 (0.847 - 1.022) | 0.132 |
| CNS tumor | 0.439 (0.209 - 0.923) | 0.03 |
| Other | 0.932 (0.705 - 1.233) | 0.623 |
| DCD | 1.062 (0.925 - 1.22) | 0.394 |
| Hight (cm) | 0.997 (0.994 - 1) | 0.079 |
| Weight (kg) | 1.001 (0.999 - 1.002) | 0.467 |
| Recipient variables |  |  |
| Age | 1.021 (1.015 - 1.026) | < 0.001 |
| Gender (F vs. M) | 0.971 (0.93 - 1.014) | 0.178 |
| Race (reference White) |  | < 0.001 |
| Black or African American | 1.172 (1.046 - 1.314) | 0.006 |
| Asian | 0.755 (0.644 - 0.883) | < 0.001 |
| Hispanic/Latino | 0.853 (0.768 - 0.948) | 0.003 |
| Other | 0.954 (0.698 - 1.305) | 0.77 |
| ABO (reference A) |  | 0.183 |
| B | 0.943 (0.841 - 1.057) | 0.31 |
| O | 1.048 (0.969 - 1.134) | 0.244 |
| AB | 0.929 (0.775 - 1.114) | 0.426 |
| Underlying liver disease (reference HCV) |  | < 0.001 |
| HBV | 0.689 (0.588 - 0.806) | < 0.001 |
| Alcohol | 0.941 (0.828 - 1.07) | 0.354 |
| NASH | 1.061 (0.931 - 1.208) | 0.376 |
| Other | 1.052 (0.934 - 1.185) | 0.401 |
| Hight (cm) | 1.003 (1 - 1.007) | 0.08 |
| Weight (kg) | 1.001 (0.999 - 1.003) | 0.561 |
| Warm ischemia time (min) | 1 (0.998 - 1.003) | 0.674 |
| Cold ischemia time (h) | 0.996 (0.983 - 1.01) | 0.586 |
| BMI | 0.998 (0.992 - 1.005) | 0.612 |
| MELD | 1.015 (1.011 - 1.019) | < 0.001 |
| Albumin (g/dl) | 0.9 (0.854 - 0.948) | < 0.001 |
| Bilirubin (mg/dl) | 1.012 (1.008 - 1.017) | < 0.001 |
| INR | 1.087 (1.042 - 1.135) | < 0.001 |
| Creatinine (mg/dl) | 1.084 (1.059 - 1.109) | < 0.001 |
| Sodium (mmol/L) | 0.992 (0.984 - 1) | 0.046 |
| Tumor Staging (beyond vs. within Milan) | 1.408 (1.15 - 1.723) | 0.001 |
| Tumor nubmer | 1.093 (1.024 - 1.166) | 0.007 |
| Largest tumor diameter (cm) | 1.058 (1.037 - 1.08) | < 0.001 |
| Sum of tumor diameters (cm) | 1.054 (1.035 - 1.073) | < 0.001 |
| AFP value (ng/ml) >400 | 1.869 (1.55 - 2.254) | < 0.001 |
| Pretransplant Treatment | 0.98 (0.896 - 1.072) | 0.663 |
| Immunosuppression Induction (reference anti-CD25) |  | 0.344 |
| Thymoglobulin | 1.091 (0.936 - 1.273) | 0.267 |
| Anti-CD25 + Thymoglobulin | 1.519 (0.679 - 3.399) | 0.309 |
| Maintenance at discharge |  |  |
| Tacrolimus | 0.842 (0.78 - 0.908) | < 0.001 |
| Cyclosporin | 1.101 (0.891 - 1.36) | 0.372 |
| Sirolimus | 1.116 (0.935 - 1.334) | 0.224 |
| Mycophenolate mofetil | 0.888 (0.827 - 0.953) | 0.001 |
| Steroids | 0.901 (0.836 - 0.972) | 0.007 |

Abbreviations: AFP, α-fetoprotein; BMI, Body mass index; CNS, Central nervous system; DCD, Donation after cardiac death; HBV, Hepatitis B virus; HCV, Hepatitis C virus; INR, International normalized ratio; MELD, Model for end-stage liver disease; NASH, Nonalcoholic steatohepatitis.

Additional file 1: Table S6. Multivariable analysis of predictors for recipient overall survival whose follow-up time was longer than 24 months

|  | HR (95% CI) | P value |
| --- | --- | --- |
| Donor age (reference Group I) |  | < 0.001 |
| Group II | 1.078 (0.971 - 1.198) | 0.159 |
| Group III | 1.12 (1.011 - 1.241) | 0.031 |
| Group IV | 1.369 (1.192 - 1.573) | < 0.001 |
| Recipient age | 1.023 (1.017 - 1.03) | < 0.001 |
| Recipient Race (reference White) |  | 0.014 |
| Black or African American | 1.145 (1.004 - 1.306) | 0.043 |
| Asian | 0.878 (0.726 - 1.062) | 0.180 |
| Hispanic/Latino | 0.884 (0.785 - 0.996) | 0.043 |
| Other | 1.158 (0.835 - 1.605) | 0.379 |
| Underlying liver diseases (reverence HCV) |  | 0.020 |
| HBV | 0.726 (0.598 - 0.881) | 0.001 |
| Alcoholic liver diseases | 0.963 (0.828 - 1.121) | 0.630 |
| NASH | 0.904 (0.771 - 1.06) | 0.214 |
| Other | 0.997 (0.868 - 1.144) | 0.962 |
| MELD | 1.012 (1.006 - 1.018) | < 0.001 |
| Recipient serum creatinine (mg/dl) | 1.053 (1.011 - 1.096) | 0.013 |
| Sum of tumor diameters (cm) | 1.045 (1.026 - 1.065) | < 0.001 |
| AFP (>= 400 ng/ml vs. < 400 ng/ml) | 1.967 (1.626 - 2.38) | < 0.001 |
| Tacrolimus maintenance at discharge | 0.872 (0.799 - 0.951) | 0.002 |
| Steroids maintenance at discharge | 0.756 (0.69 - 0.828) | < 0.001 |

Abbreviations: AFP, α-fetoprotein; HBV, Hepatitis B virus; HCV, Hepatitis C virus; MELD, Model for end-stage liver disease; NASH, Nonalcoholic steatohepatitis.
