# Supplementary figures and images for: Impact of donor age on liver transplant outcomes in patients with hepatocellular carcinoma: analysis of the SRTR database
Source: BMC Gastroenterol. 2021 Apr 30;21:195. doi: 10.1186/s12876-021-01786-6 (PMC8086097; doi:10.1186/s12876-021-01786-6)

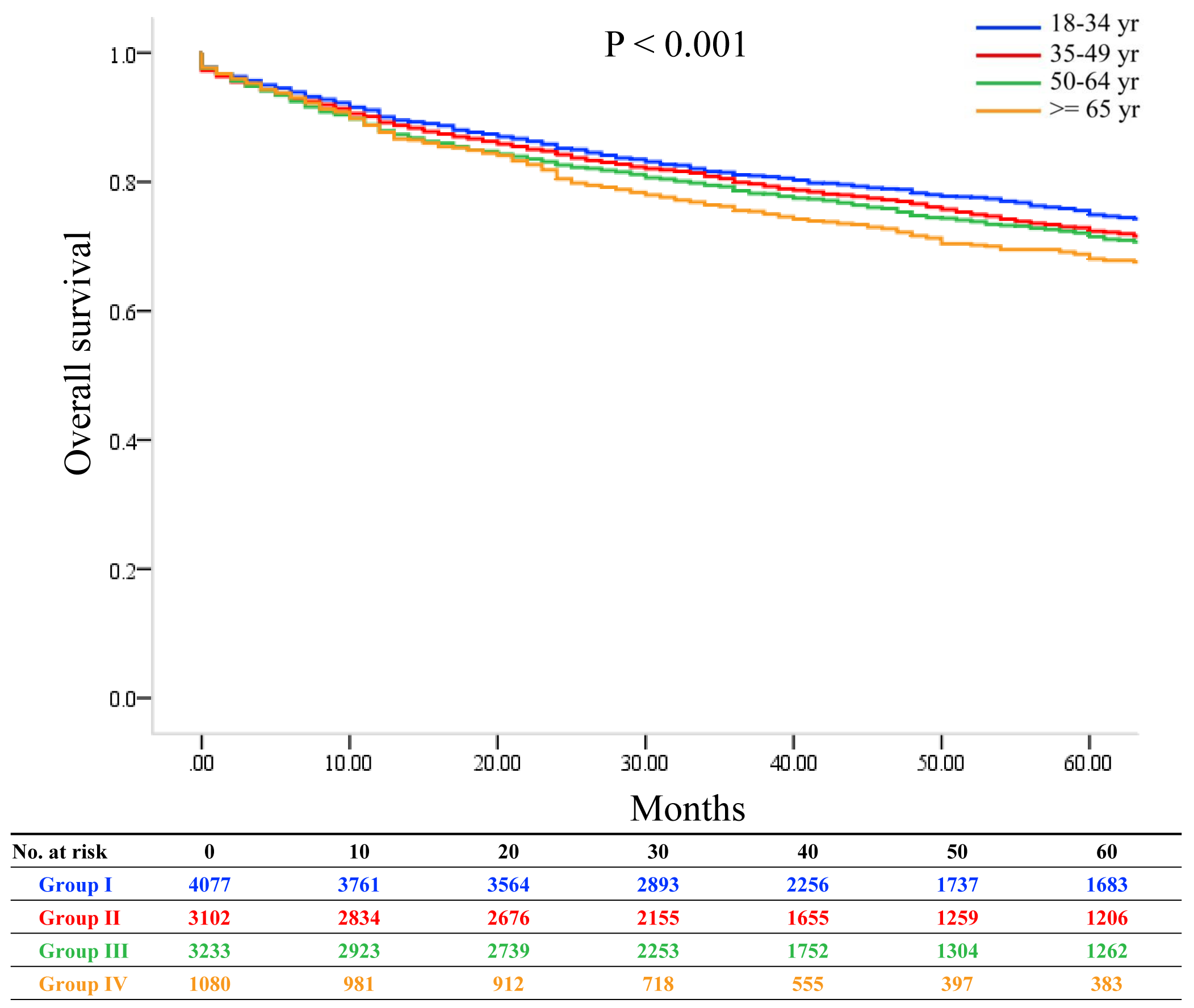

Supplement: Supplementary file 2 — Additional file 2: Figure S1. Overall survival of HCC recipients with post-transplant follow-up time ≥ 24 months categorized by different donor age groups. [file 12876_2021_1786_MOESM2_ESM.tif]

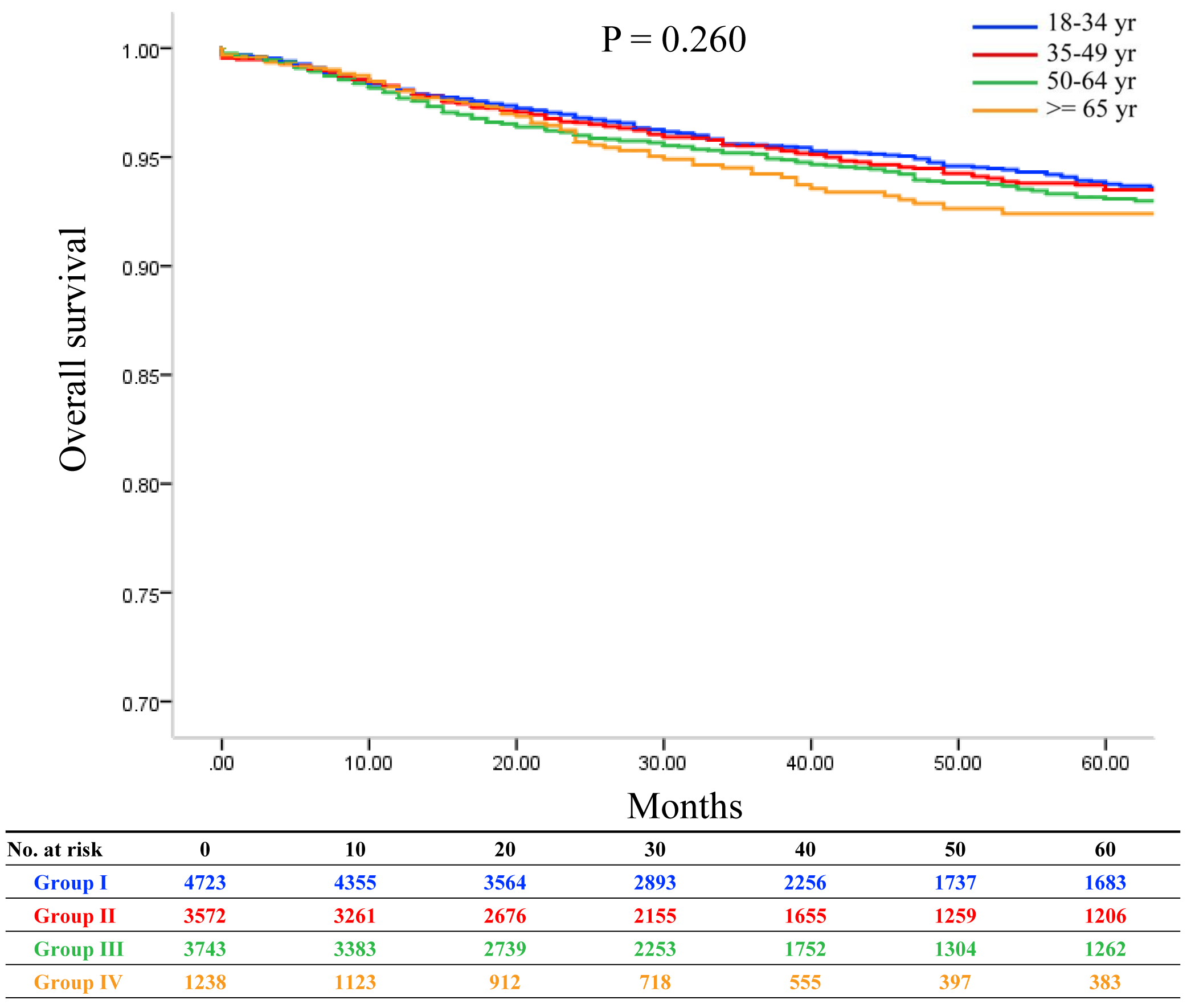

Supplement: Supplementary file 3 — Additional file 3: Figure S2. Liver-specific survival of HCC recipients categorized by different donor age groups. [file 12876_2021_1786_MOESM3_ESM.tif]

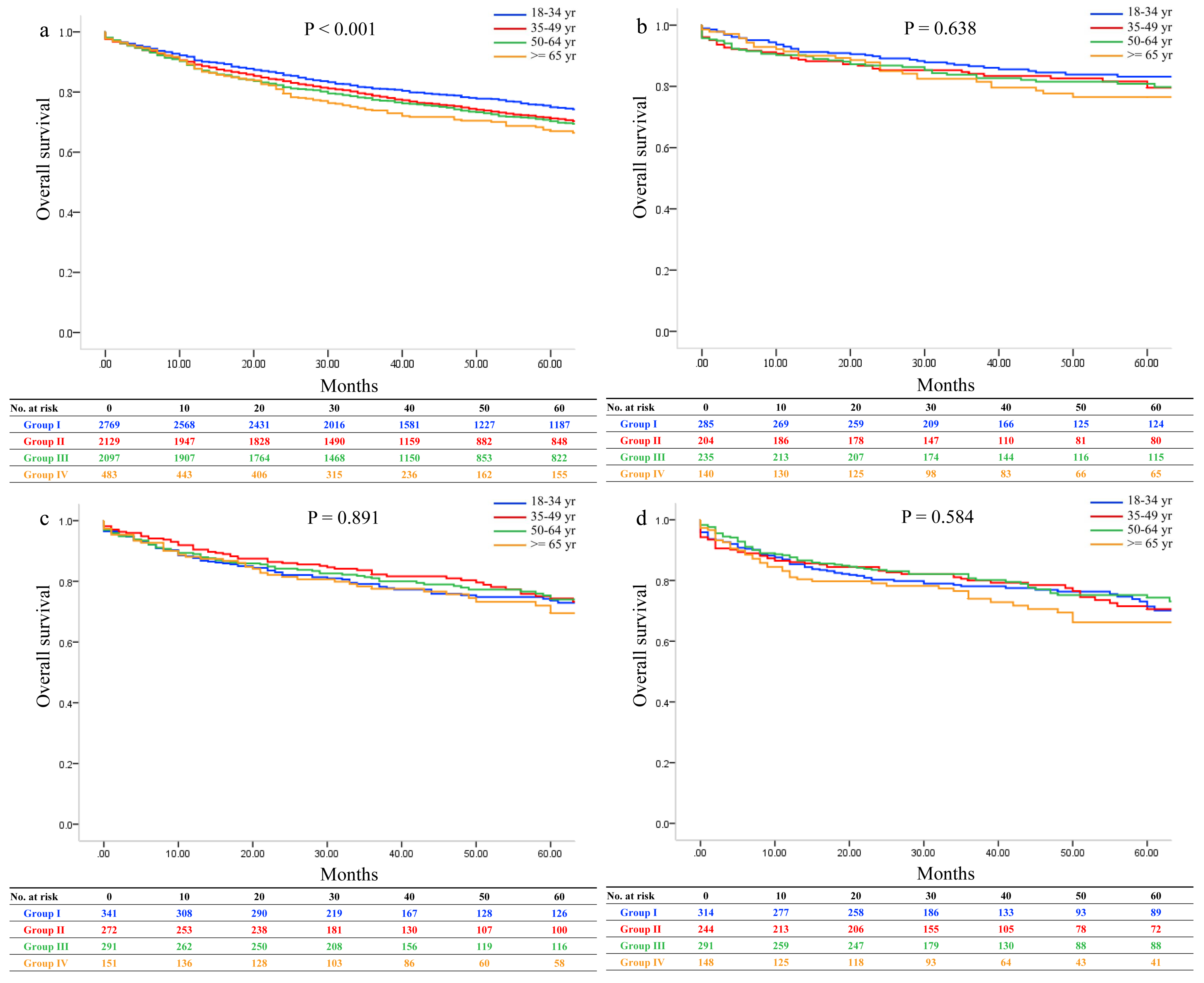

Supplement: Supplementary file 4 — Additional file 4: Figure S3. Overall survival of HCC recipients with post-transplant follow-up time ≥ 24 months in different donor age groups according to underlying liver diseases: a HCV; b HBV; c alcoholic liver diseases; d NASH. [file 12876_2021_1786_MOESM4_ESM.tif]

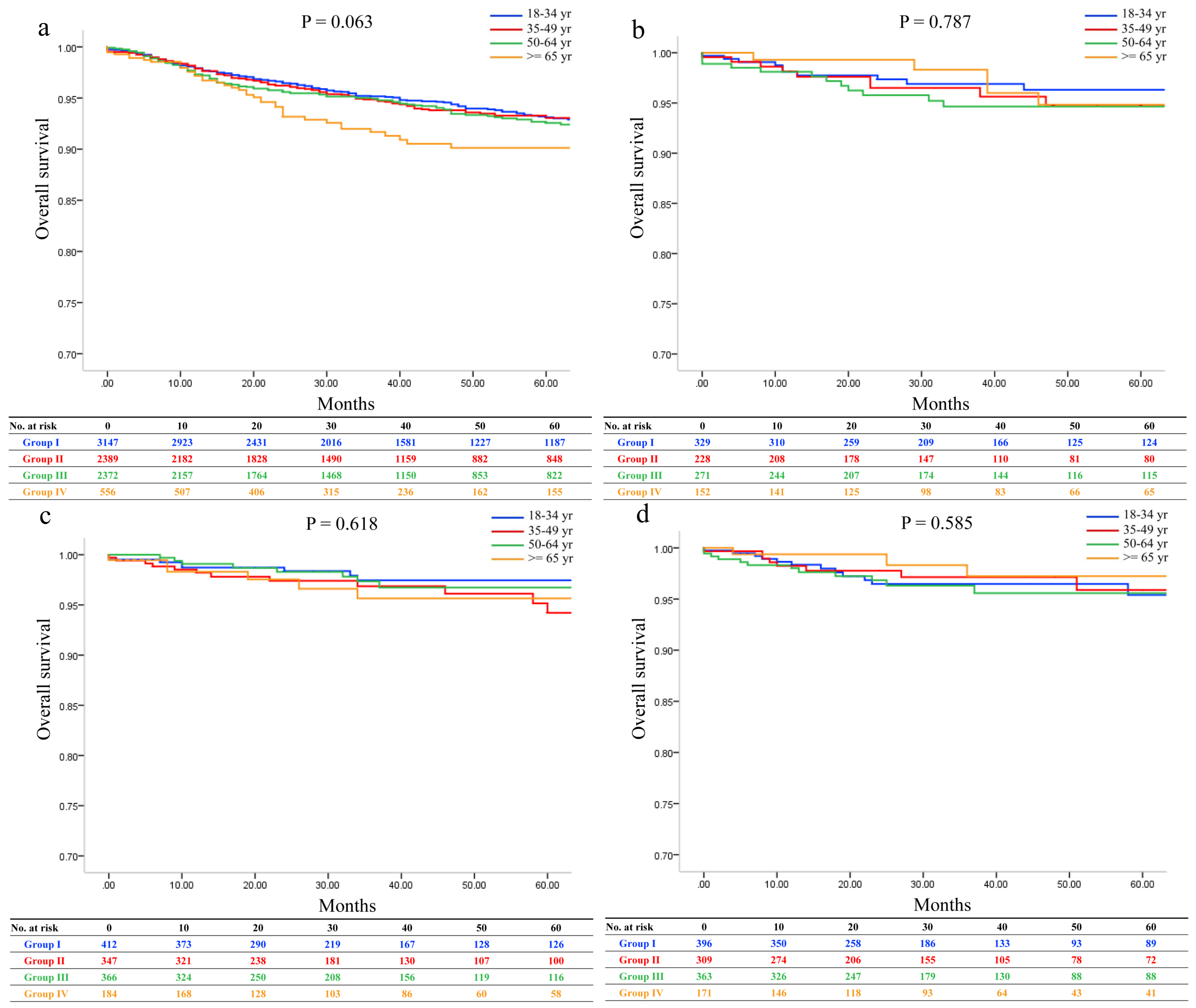

Supplement: Supplementary file 5 — Additional file 5: Figure S4. Liver-specific survival of HCC recipients in different donor age groups according to underlying liver diseases: a HCV; b HBV; c alcoholic liver diseases; d NASH. [file 12876_2021_1786_MOESM5_ESM.tif]

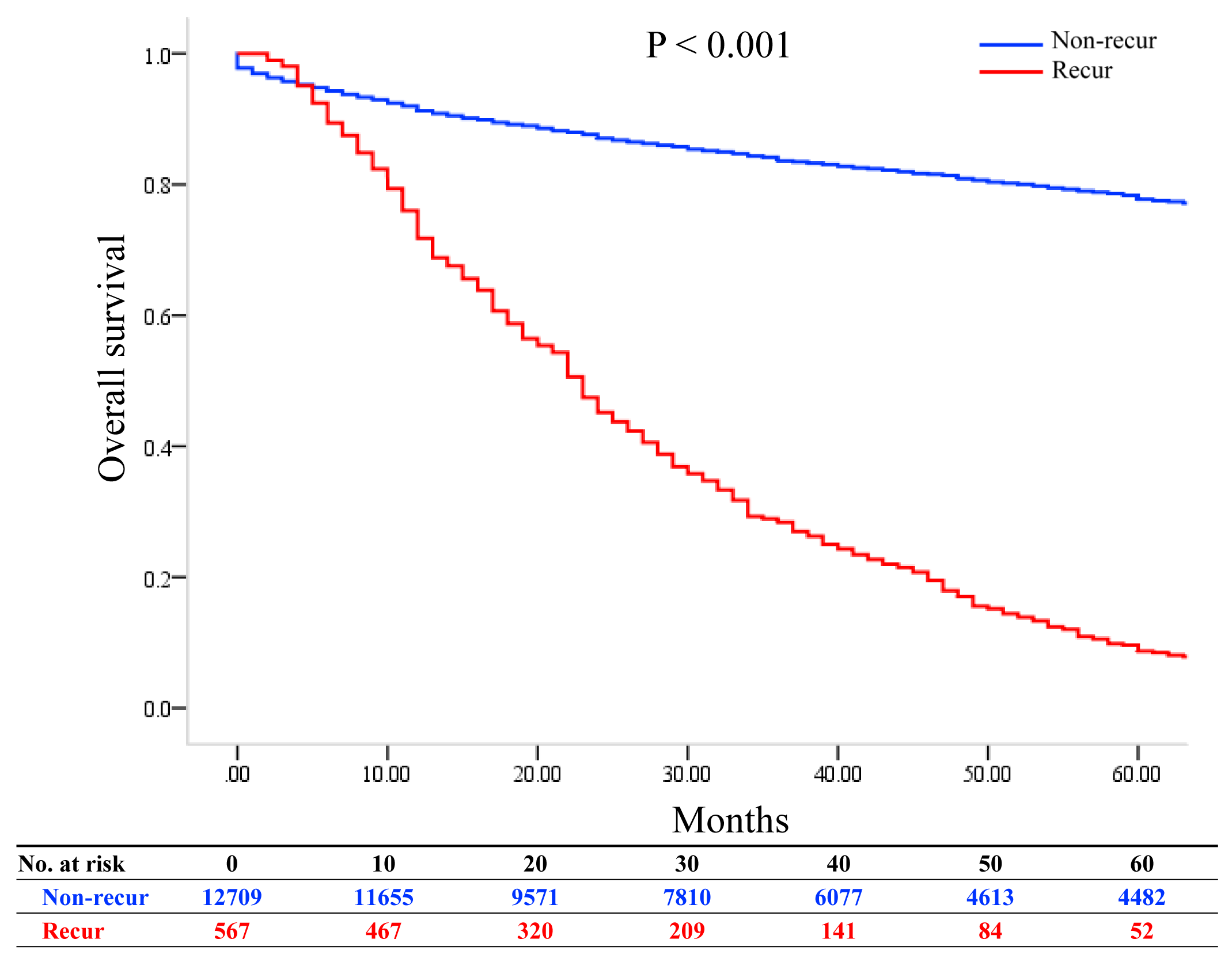

Supplement: Supplementary file 6 — Additional file 6: Figure S5. Overall survival between HCC recipients with and without post-transplant tumor recurrence. [file 12876_2021_1786_MOESM6_ESM.tif]

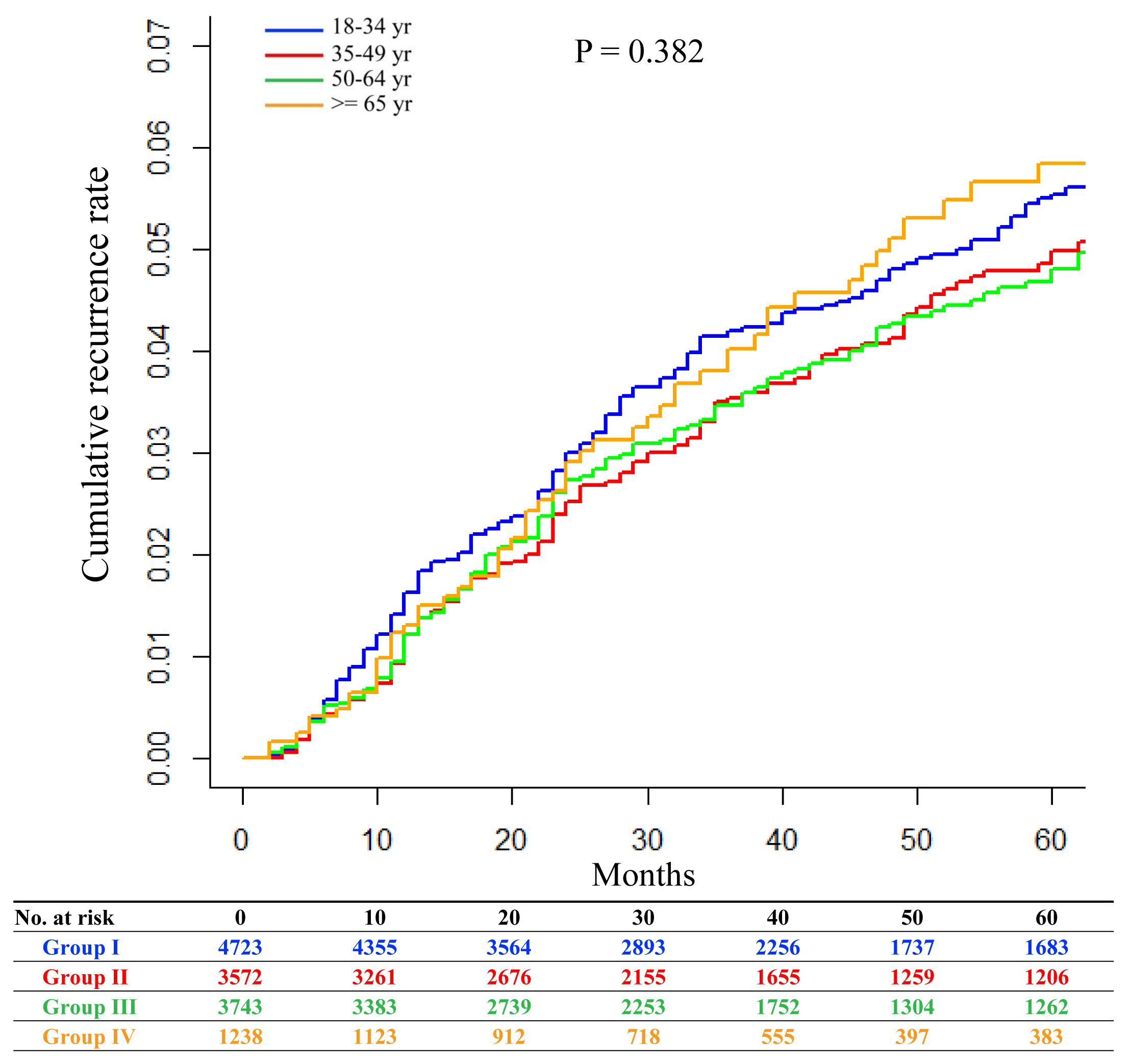

Supplement: Supplementary file 7 — Additional file 7: Figure S6. Cumulative HCC recurrence rates categorized by different donor age groups. [file 12876_2021_1786_MOESM7_ESM.tif]

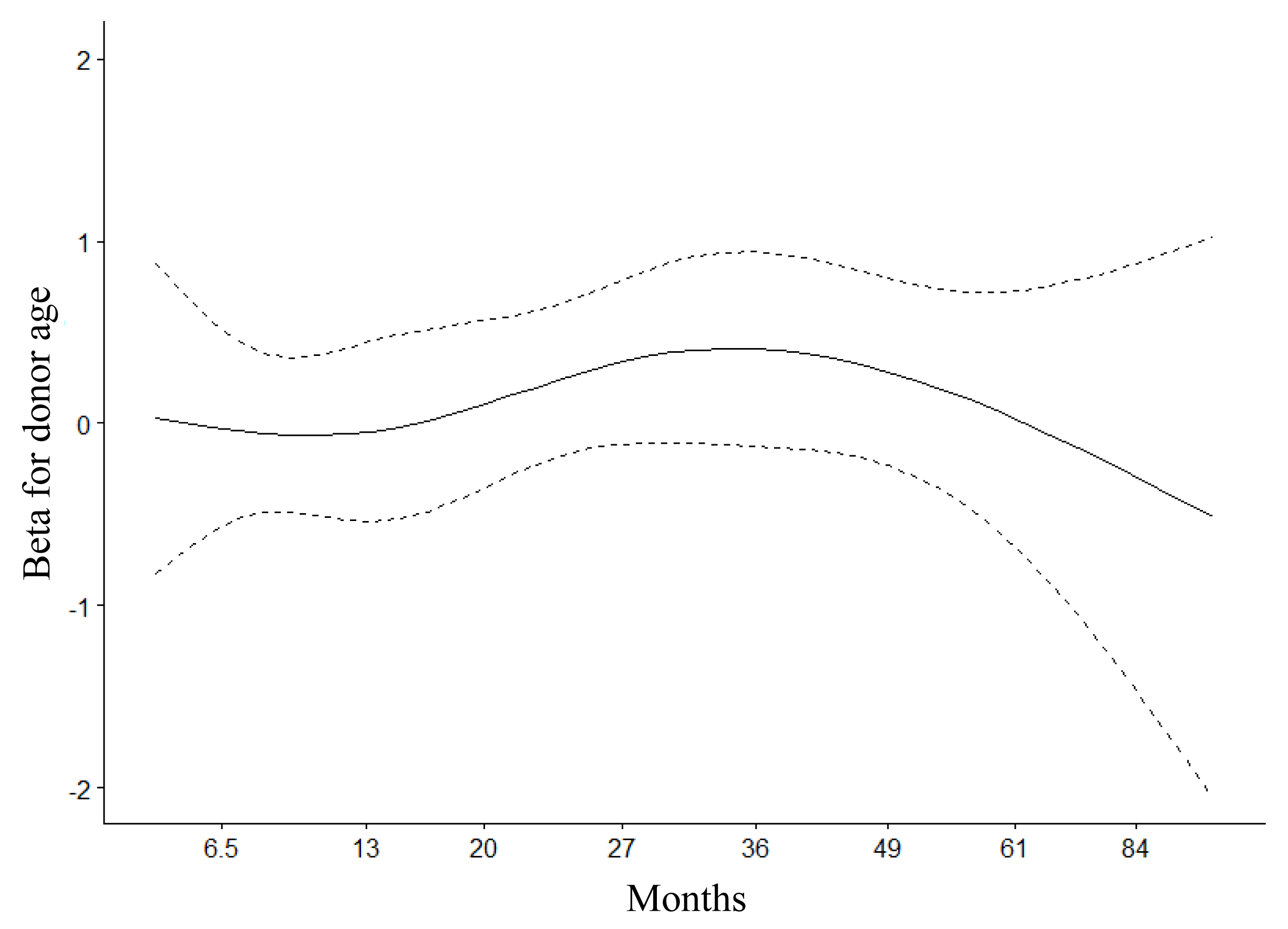

Supplement: Supplementary file 8 — Additional file 8: Figure S7. Cox-derived estimates of the time-dependent hazard ratio of donor age for HCC recurrence after liver transplantation. [file 12876_2021_1786_MOESM8_ESM.tif]
